# Genetic Dissection and Validation of Chromosomal Regions for Transmission Ratio Distortion in Intersubspecific Crosses of Rice

**DOI:** 10.3389/fpls.2020.563548

**Published:** 2020-10-27

**Authors:** Chaopu Zhang, Dianwen Wang, Jilin Wang, Qiang Sun, Li Tian, Xinxin Tang, Zhiyang Yuan, Hanzi He, Sibin Yu

**Affiliations:** ^1^National Key Laboratory of Crop Genetic Improvement, Huazhong Agricultural University, Wuhan, China; ^2^College of Plant Science and Technology, Huazhong Agricultural University, Wuhan, China

**Keywords:** rice, transmission ratio distortion, backcross inbred lines, chromosome segment substitution line, allele frequency, epistatic interaction

## Abstract

Transmission ratio distortion (TRD) refers to a widespread phenomenon in which one allele is transmitted by heterozygotes more frequently to the progeny than the opposite allele. TRD is considered as a mark suggesting the presence of a reproductive barrier. However, the genetic and molecular mechanisms underlying TRD in rice remain largely unknown. In the present study, a population of backcross inbred lines (BILs) derived from the cross of a *japonica* cultivar Nipponbare (NIP) and an *indica* variety 9311 was utilized to study the genetic base of TRD. A total of 18 genomic regions were identified for TRD in the BILs. Among them, 12 and 6 regions showed *indica* (9311) and *japonica* (NIP) alleles with preferential transmission, respectively. A series of F_2_ populations were used to confirm the TRD effects, including six genomic regions that were confirmed by chromosome segment substitution line (CSSL)-derived F_2_ populations from intersubspecific allelic combinations. However, none of the regions was confirmed by the CSSL-derived populations from intrasubspecific allelic combination. Furthermore, significant epistatic interaction was found between *TRD1.3* and *TRD8.1* suggesting that TRD could positively contribute to breaking intersubspecific reproductive barriers. Our results have laid the foundation for identifying the TRD genes and provide an effective strategy to breakdown TRD for breeding wide-compatible lines, which will be further utilized in the intersubspecific hybrid breeding programs.

## Introduction

Reproductive isolation is recognized as a powerful driving force for maintaining species identity (Ouyang et al., 2010; Dagilis et al., 2018). An important genetic factor maintaining reproductive isolation is transmission ratio distortion (TRD), which is defined as the allele inheritance in progenies of hybrids that show a statistically significant deviation from the expected Mendelian segregation ratios, also leading to deviations in genotype frequencies (Koide et al., 2012; Leppala et al., 2013; Li et al., 2019). TRD acts as one of the selfish genetic elements, distorting segregation or non-Mendelian transmission of alleles that can alter allele transmission in hybrids among progenies of heterozygotes, since the gametes carrying competing alleles increase their transmission over other alleles (Masly et al., 2006; Moyle and Graham, 2006; Koide et al., 2008a; Phadnis and Orr, 2009; Phadnis, 2011). TRD has been described in intersubspecific and intrasubspecific hybrids in various animal and plant species, such as *Drosophila* (Mcdermott and Noor, 2012; Corbett-Detig et al., 2013), mouse (Bauer et al., 2005, 2007), mosquito (Shin et al., 2012), rice (Yang et al., 2012; Reflinur et al., 2014; Shen et al., 2017; Xie et al., 2019), *Arabidopsis* (Bikard et al., 2009; Leppala et al., 2013), and lettuce (Giesbers et al., 2019).

Multiple genetic factors could affect TRD such as chromosome segregation during meiosis (Fishman and Willis, 2005; Fishman and Sweigart, 2018) as well as prezygotic and postzygotic isolation (Harushima et al., 2001; Koide et al., 2008b). In addition, environmental fluctuations can result in allelic frequency changes at a single locus. For example, allelic frequency can change as conditions shift from drought to rainfall (Allard et al., 1992). Multiple loci have been proposed to explain reproductive isolation (Sano, 1990; Orr, 1996; Orr and Turelli, 2001; Phadnis and Orr, 2009). Several well-known reproductive isolation systems have been identified and characterized (Kusano et al., 2001; Kusano et al., 2002; Hu et al., 2017). For example, in mouse (*Mus musculus* L.), the *t* haplotype system including the *t* complex responder (*Tcr*) and the *t* complex distorter (*Tcd*) genes correspond to reduced fertility, and preferentially transmit the *Tcr* (*t*-haplotype) alleles in the heterozygous male progeny (Schimenti, 2000; Willison and Lyon, 2000). In *Drosophila* (*Drosophila melanogaster*), the segregation distorter (*SD*) system containing the distorter *SD/SD* + causes preferential transmission of male alleles due to the dysfunction of *SD* + spermatids (Merrill et al., 1999; Kusano et al., 2001, 2002). Three genes in fission yeast (*Schizosaccharomyces kombucha* and *Schizosaccharomyces pombe*), *cw9*, *cw27*, and *wtf4*, from the self-driver *wtf* (for with *Tf*) gene family encode both a gamete-killing poison and an antidote to the poison such that non-protected *S. pombe* (*Sp*) gametes are killed, and thus there is a loss of transmission of the *Sp* allele from heterozygotes (Hu et al., 2017; Nuckolls et al., 2017).

In rice, the *S5* killer-protector system includes three tightly linked genes on chromosome 6 that regulate both segregation distortion and hybrid embryo-sac fertility. *ORF4*+ and *ORF5*+ constituted a killer system that could kill the female gametes with *ORF3*−, resulting in distorted allele frequency in heterozygotes, but the *ORF3*+ allele could rescue these alleles (Yang et al., 2012). Recently, a toxin–antidote system of *qHMS7* was identified for male sterility that contains two tightly linked genes, *ORF2* and *ORF3*, encoding toxic and antidote genetic elements, respectively (Yu et al., 2018). The African rice *S1* allele (*S1-g*) including three closely linked genes (*S1A4*, *S1TPR*, and *S1A6*) that constitute a killer-protector system can eliminate gametes carrying the Asian allele (*S1-s*), leading to significant preferential transmission of the *S1-g* allele (Xie et al., 2019). Overall, these findings indicate that the genetic and molecular mechanisms regulating TRD are involved in allelic interactions at either a single locus or multiple loci. Therefore, it is critical to identify the genomic regions for TRD since it is the first step toward dissecting the molecular basis of such a complex trait.

The *indica* and *japonica* are the two major subspecies of Asian cultivated rice (*Oryza sativa* L.) (Huang et al., 2012). Reproductive barriers such as hybrid incompatibility and hybrid weakness are commonly observed in crosses between *indica* and *japonica*, and intersubspecific hybrids have more serious reproductive isolation than intrasubspecific crosses (Ouyang and Zhang, 2013). Meanwhile, reproductive isolation causes poor utilization of heterosis in hybrid rice (Ouyang et al., 2010). The objective of the present study is to identify the TRD regions to investigate the genetic basis of TRD in rice. We conducted TRD analyses by using backcross inbred lines (BILs), which were derived from an intersubspecific cross of the *japonica* cultivar Nipponbare and the *indica* variety 9311. As a result, a number of genomic regions were identified for TRD in the intersubspecific mapping BILs and validated in CSSL-derived F_2_ populations. Furthermore, interaction effects of two regions on TRD were explored. These findings could provide new insights into the genetic basis of TRD in rice.

## Materials and Methods

### BIL Population and CSSL-Derived Populations

The BIL population and chromosome segment substitution line (CSSL-derived) population were used to dissect the genetic basis of TRD in rice. The BILs, which included 437 lines, were developed by single-seed descent from the cross between two genome-sequenced rice cultivars, the *japonica* variety Nipponbare (NIP) and the elite *indica* restorer line 9311 (Yuan et al., 2019). Briefly, NIP (male) was crossed with 9311 (female), and the F_1_ was then backcrossed to 9311 (female) to obtain BC_1_F_1_ lines. Subsequently, 437 BC_1_F_8_ lines were produced by the single-seed descent method and were genotyped for TRD analysis. To validate the effect of the TRD regions, three types of CSSLs were derived using the backcross scheme with marker-assisted selection approach (Sun et al., 2015; Zhang et al., 2020). First, 12 CSSLs, each carried the introduced NIP segment surrounding the TRD region of interest within the 9311 background, were developed by using NIP (male) as the donor and 9311 (female) as the recurrent parent, named as NY lines. Second, nine CSSLs in the ZS97 background were developed using NIP (male) as the donor and an *indica* cultivar Zhenshan 97 (ZS97, female) as the recurrent parent, then named as NZ lines. Third, five CSSLs each carrying the introduced MH63 segment in the ZS97 background were developed using an *indica* Minghui 63 (MH63, male) as the donor and ZS97 (female) as the recurrent parent, named as MZ lines. These 26 CSSLs were then crossed with the recurrent parent (9311 or ZS97) to generate 26 relevant F_2_ populations (hereafter named as the NY-, NZ-, and MZ-derived populations, respectively). The other line harboring two introduced NIP segments surrounding *TRD1.3* and *TRD8.1* was used to generate an additional F_2_ population. The BIL population and CSSLs were grown at the experimental field of Huazhong Agricultural University (HAU) in Wuhan (30.48N, 114.2E), China, and each line was planted in a row with 10 individual spacings of 16.7 × 26.6 cm. The CSSL-derived F_2_ populations, comprising a various number of individuals were planted in the same field and used for genotype analysis. Field management was carried out according to the local standard practices.

### Genotype Analyses

Genomic DNA was extracted from young seedling leaves using the CTAB method as described previously (Murray and Thompson, 1980). The 437 BILs were genotyped using a genotyping-by-sequencing (GBS) strategy (Yuan et al., 2019). The GBS library were generated as described in a previous report (Elshire et al., 2011), where the genomic DNA from each of the BILs was digested with *Mse*I, followed by end blunting, dATP addition at the 3′ end, and ligation of Y-adapters. Fragments of 300–550 base pairs (bp) in size were isolated using a Gel Extraction kit (Qiagen, Valencia, CA, United States). These fragments were then purified using the Agencourt AMPure XP System, then diluted for sequencing. Sequencing was performed on the selected tags using Illumina HiSeq (Illumina, San Diego, CA, United States) with the paired-end mode and 150-bp read length (Huang et al., 2009; Elshire et al., 2011). The Burrows Wheeler Aligner software (V0.7.15) was used to map the clean reads for each sample on the reference genome (MSU7.0^1^) (Li and Durbin, 2009). Single-nucleotide polymorphism (SNP) calling was performed by Genome Analysis Toolkit (McKenna et al., 2010). In total, 49,890 high-quality SNPs were identified for the BILs after filtering out the low-quality SNPs using three criteria: (1) minor allele frequency ≥ 5%, (2) heterozygous genotype ≤ 20%, and (3) missing genotype ≤ 20%. The heterozygous genotypes were set as missing data. Due to 37 lines with >20% missing data, a high-density bin map was generated based on SNP genotypes of 400 BILs as previously described with a minor modification (Huang et al., 2009; Li et al., 2017). Briefly, the genotype of each line was scanned with a sliding window of 15 SNPs and a step size of 1. An “a/b” ratio of 12:3 or higher was recognized as “a,” and 3:12 or lower was recognized as “b.” The missing genotypes were coded as “−”. Adjacent windows with the same genotype were combined into a block, and the recombinant breakpoint information was assumed to be at the boundary of adjacent blocks with different genotypes. The interval between two adjacent crossovers in the entire population was defined as a recombination bin. A genetic linkage bin map was constructed for further analysis using the “MAP” function in IciMapping (Li et al., 2007). The insertion/deletion (Indel) and simple sequence repeat (SSR) polymorphic markers that could separate the paired parents (ZS97 vs. NIP, 9311 vs. NIP, ZS97 vs. MH63) were selected to genotype the CSSL-derived populations. To determine the digenic interaction between *TRD1.3* and *TRD8.1*, two polymorphic markers, SD1C and SD8C, that were linked with *TRD1.3* and *TRD8.1*, respectively, were used to classify the nine genotypes in the F_2_ population. All the Indel and SSR markers were separated using 4% polyacrylamide gel electrophoresis and silver stains for visualization. The bin genotypes of the BILs are provided in Supplementary Table S1.

### Transmission Ratio Distortion Analysis

For BILs, the genotype and allele frequencies are the same values for each bin since the BILs had only two homozygous genotypes (NIP and 9311). Deviation of observed allele frequency of each bin in the BILs from the theoretical Mendelian segregation ratio (3:1 for both allele and genotype frequency) was investigated by a chi-square test. The chi-square (χ^2^) and *P*-values were determined by using R function *chisq.test*^2^. The bin showing non-Mendelian segregation (*P* < 0.0001) was initially considered to have a significant TRD effect. The TRD region analysis in the BILs was performed using the single-marker analysis (SMA) model in QTL IciMapping (v4.0) (Li et al., 2007). A significance level of LOD > 4.5 was set as the threshold to declare the presence of the putative TRD effect in a given bin. The nomenclature of the TRD region or locus followed the principles suggested by a previous report (McCouch, 2008). When several adjacent bins exhibited TRD effects with the same allele transmission pattern, a TRD region was presented with peak bin and its interval defined as the first and last bins that showed TRD effects (Li et al., 2017, 2019). The statistical χ^2^ and *P*-values of the observed allele and genotype frequency of each polymorphic marker distorted from the expected segregation ratios in the CSSL-derived populations, and nine genotype ratios in the additional F_2_ population were also determined by using R function *chisq.test*.

## Results

### Bin Markers Displaying Transmission Ratio Distortion

The BIL population (*n* = 400) was used to evaluate the genetic basis of TRD. The high-density bin map was generated with 3,235 bins as markers, which were evenly distributed on all of the 12 chromosomes, and the bin lengths ranged from 30 kb to 3.0 Mb with an average of 115 kb in these 400 BILs. There were 745 out of 3,235 bins showing significant distortion from the expected Mendelian allele segregation ratio (3:1), which were distributed on all of the chromosomes except for chromosomes 7, 9, 10, and 11 (Supplementary Figure S1 and Supplementary Table S2). Among all the distorted markers, 179 and 566 markers were significantly skewed toward NIP and 9311, respectively.

### Detection of Genomic Regions for Transmission Ratio Distortion in Backcross Inbred Lines

**FIGURE 1:**
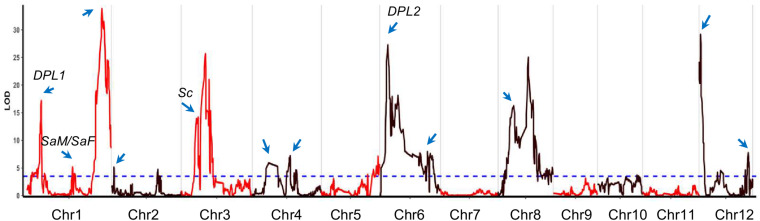
The transmission ratio distortion (TRD) regions identified in backcross inbred lines (BILs). The *x*-axis represents the physical location along each numbered chromosome. The *y*-axis represents the logarithm of odds (LOD) values. Horizontal dashed line indicates the declaration threshold. Blue arrows represent the regions or genes associated with TRD or segregation distortion in previous studies. Four reported genes are highlighted.

To detect the distorted genomic regions more precisely, the SMA method in QLT IciMapping was conducted in the BILs (Li et al., 2007). A total of 18 genomic regions (91.9 Mb, approximately 24.8% of genome coverage based on the Nipponbare genome) were identified for TRD in the BILs (Figure 1 and Supplementary Table S3). Among them, chromosomes 1, 8, and 12 each contained three TRD loci or regions, whereas one region was identified on chromosome 5. Twelve and six regions with the alleles were significantly skewed toward 9311 and NIP, respectively (Supplementary Table S3). These results were consistent with the allele segregation ratios determined by using bin markers in the BILs.

Among the identified TRD regions, *TRD1.3* in Bin390 (38.3–38.4 Mb) on chromosome 1 had the most significant effect (LOD = 33.8; χ^2^ = 179.4) and was significantly skewed toward the NIP allele (Supplementary Table S3). Four regions (*TRD3.2*, *TRD6.1*, *TRD8.2*, and *TRD12.1*) showed a moderate effect (LOD ≥ 20.0), and all of them were significantly skewed toward 9311. For the remaining 13 regions with relative minor effects (LOD < 20.0), eight regions (*TRD1.1*, *TRD1.2*, *TRD3.1*, *TRD4.1*, *TRD6.2*, *TRD8.1*, *TRD8.3*, and *TRD12.3*) in which alleles were significantly skewed toward 9311, and five regions (*TRD2.1*, *TRD2.2*, *TRD4.2*, *TRD5*, and *TRD12.2*) were significantly skewed toward NIP (Supplementary Table S3). These data indicate that 9311 gametes of most TRD regions carried competing alleles, thus gaining a transmission advantage over NIP alleles in the BILs.

### Validation of the Transmission Ratio Distortion Regions in Chromosome Segment Substitution Line-Derived Populations

To validate the TRD effects, a total of 12 CSSLs (NY) that each carried a particular introduced NIP segment (*TRD1.2*, *TRD1.3*, *TRD2.1*, *TRD2.2*, *TRD3.1*, *TRD3.2*, *TRD4.1*, *TRD5*, *TRD6.2*, *TRD8.1*, *TRD8.3*, or *TRD12.2*) were selected and crossed with 9311 to produce the 12 NY-derived populations (Supplementary Table S4). Meanwhile, nine CSSLs that each carried the introduced NIP segment encompassing a corresponding locus (*TRD1.3*, *TRD2.1*, *TRD2.2*, *TRD3.1*, *TRD3.2*, *TRD4.1*, *TRD5*, *TRD8.1*, or *TRD12.2*) were crossed with ZS97 to produce the relevant NZ-derived populations (Supplementary Table S5). Five CSSLs that each carried the introduced *indica* MH63 segment harboring a target locus (*TRD1.2*, *TRD2.1*, *TRD3.2*, *TRD8.1*, or *TRD12.2*) were also crossed with ZS97 to produce the relevant intrasubspecific MZ-derived populations (Supplementary Table S6). In addition, 2–11 polymorphic SSRs and Indel markers (Supplementary Table S7) that were evenly distributed in every relevant TRD region were used to investigate the genotype of the F_2_ populations.

**FIGURE 2:**
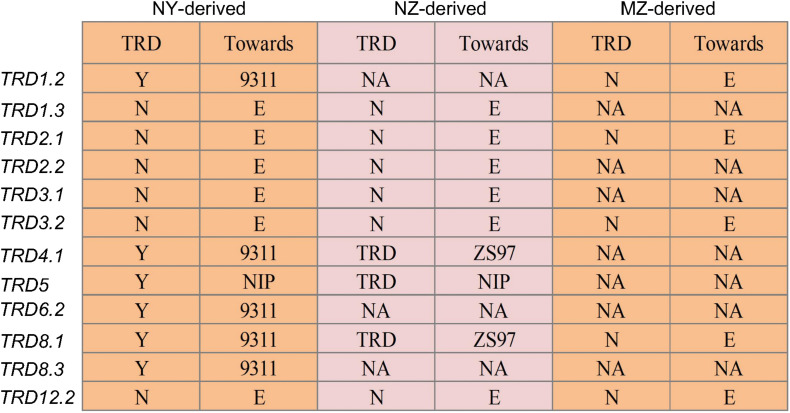
Validation of 12 genomic regions for TRD in the chromosome segment substitution line (CSSL)-derived F_2_ populations. NY-derived and NZ-derived indicate the populations developed using Nipponbare (NIP) as the donor in 9311 and Zhenshan 97 (ZS97) backgrounds, respectively. MZ-derived represent the F_2_ population developed using Minghui 63 (MH63) as the donor in the ZS97 background. “N” and “Y” indicate the absence and presence of TRD effect in a given region, respectively. “Toward” indicates that the allele in the heterozygote was preferentially transmitted to progeny. “E” indicates that the two alleles in the heterozygote were transmitted equally to progeny. NA, data not available.

**FIGURE 3:**
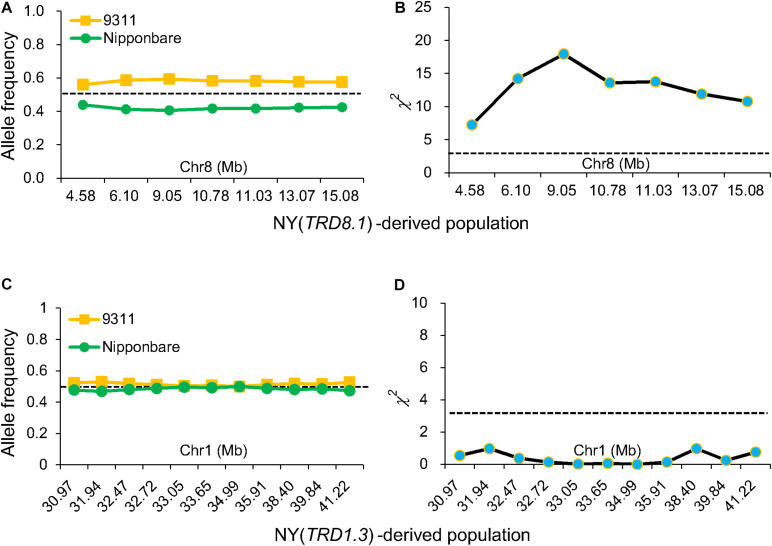
Validation of *TRD1.3* and *TRD8.1* effects on TRD. Allele frequencies **(A)** and chi-square (χ^2^) test for allele frequencies **(B)** at seven markers in the *TRD8.1* region in the NY(*TRD8.1*)-derived population (*n* = 272). Allele frequencies **(C)** and chi-square test for allele frequencies **(D)** at 11 markers in the *TRD1.3* region in the NY (*TRD1.3*)-derived population (*n* = 131). Horizontal dashed lines indicate the theoretical segregation ratio (1:1) in **(A,C)** and the significant threshold at *P* < 0.05 in **(B,D)**. The *x*-axis represents the physical locations of the used markers along the numbered chromosome.

TRD analysis in the 12 NY-derived populations confirmed that six regions (*TRD1.2*, *TRD4.1*, *TRD5*, *TRD6.2*, *TRD8.1*, and *TRD8.3*) exhibited a significant effect on TRD (Figure 2). For example, the NY(*TRD1.2*)-derived population (*n* = 188) was genotyped using seven polymorphic markers that were evenly distributed in the *TRD1.2* region to validate the TRD effect (Figure 2 and Supplementary Table S4). Six out of seven markers, including ID01C061, ID01C063, ID01C065, ID01C067, ID01C068, and ID01C071 were significantly distorted from both the expected ratio (1:2:1) for genotype frequency and the expected ratio (1:1) for allele frequency. These six distorted markers were all skewed toward 9311 (Supplementary Table S4), indicating that the 9311 alleles at *TRD1.2* were transmitted to the progeny at higher frequencies than NIP alleles in heterozygotes. The NY (*TRD8.1*)-derived population (*n* = 272) was genotyped using seven polymorphic markers, and these markers were all significantly distorted from the expected Mendelian genotype and allele segregation ratios, and skewed toward 9311 (Figures 2, 3 and Supplementary Table S4). Using the same approach, *TRD4.1*, *TRD5*, *TRD6.2*, and *TRD8.3* effects were also confirmed in the relevant NY-derived populations. The genotypes and alleles at the three regions (*TRD4.1*, *TRD6.2*, and *TRD8.3*) were all skewed toward 9311, and the NIP alleles had preferential transmission at *TRD5*, leading to the genotype skewed toward NIP in the progeny (Figure 2 and Supplementary Table S4). Thus, five TRD regions (*TRD1.2*, *TRD4.1*, *TRD6.2*, *TRD8.1*, and *TRD8.3*) in which the alleles were significantly skewed toward 9311, indicating that the 9311 alleles were transmitted to the progeny at higher frequencies than NIP alleles in the F_2_ populations.

Three regions (*TRD4.1*, *TRD5*, and *TRD8.1*) also exhibited significant effects on TRD in NZ-derived populations (Figure 2). *TRD4.1* and *TRD8.1* were both confirmed in NY-derived and NZ-derived populations. Nine assayed markers at *TRD4.1* were all skewed toward *indica* alleles (9311 or ZS97). The TRD effect of *TRD8.1* was also validated in the NZ (*TRD8.1*)-derived population (*n* = 175), and the markers within the region were significantly skewed toward ZS97, while the *japonica* (NIP) alleles at *TRD5* were transmitted to the progeny at higher frequencies than either 9311 or ZS97 alleles (Figure 2 and Supplementary Table S5). These results indicated that either 9311 or ZS97 alleles at *TRD4.1* and *TRD8.1* revealed preferential transmission to the progeny over the NIP alleles (Figure 2 and Supplementary Tables S4, S5).

Meanwhile, five regions (*TRD1.2*, *TRD2.1*, *TRD3.2*, *TRD8.1*, and *TRD12.2*) with TRD effects detected in the BILs were not validated in the MZ-derived populations (Figure 2). Three regions of *TRD2.1*, *TRD3.2*, and *TRD12.2* had no effects on TRD in either NY-derived population or NZ-derived population (Figure 2 and Supplementary Table S6). Consistently, these distorted regions were not detected in the previous study with an F_2_ population derived from the same combination of MH63 and ZS97 (Li et al., 2017).

### Epistatic Interaction Effect Between *TRD1.3* and Other Genomic Regions

**FIGURE 4:**
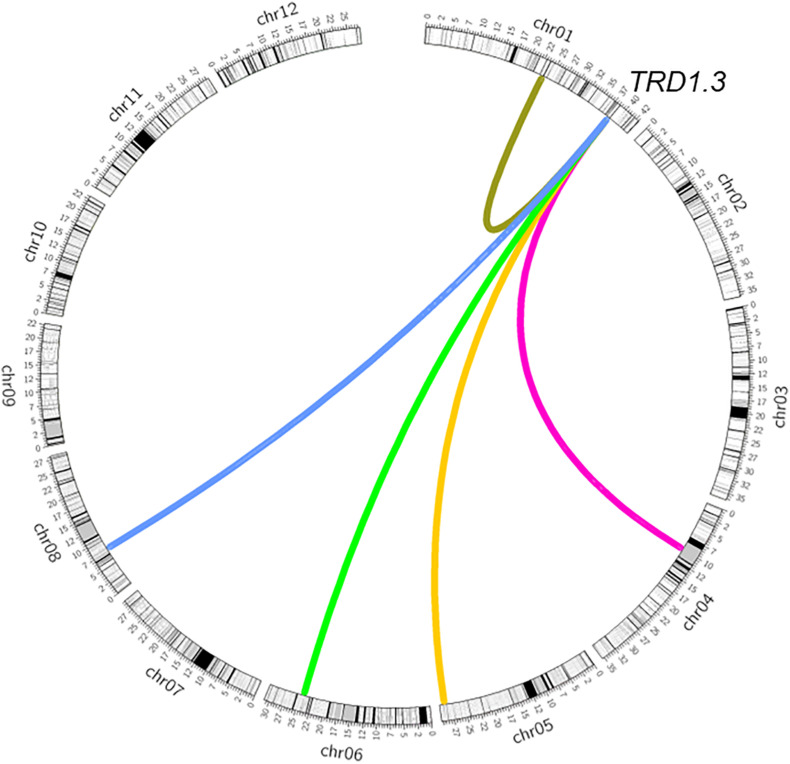
Epistatic interaction of *TRD1.3* with other chromosomal regions affecting transmission ratio distortion. Rice chromosomes with bins are indicated in the outer circle. The colored connection lines represent the epistatic interactions of *TRD1.3* with the regions (*TRD1.2*, *TRD4.1*, *TRD5*, *TRD6.2*, *TRD8.1*).

To validate the *TRD1.3* effect in the BILs, NY(*TRD1.3*)-derived population (*n* = 131) and NZ (*TRD1.3*)-derived population (*n* = 157) were used (Supplementary Tables S4, S5). Unexpectedly, all assayed markers in the *TRD1.3* region showed normal-Mendelian segregation ratios in both F_2_ populations (Figures 3C,D). These results indicated that the *TRD1.3* effect was dependent on other loci within the backgrounds of 9311 or ZS97. To detect the regions that interacted with *TRD1.3*, the BILs were separated into two subpopulations that had a fixed NIP (named as SubN, *n* = 172) or 9311 genotypes (named as SubY, *n* = 181) at the *TRD1.3* region, respectively. The other 47 lines were removed due to missing data (*n* = 17) and heterogeneous recombination (*n* = 30) in the *TRD1.3* region. A total of 14 genomic regions were identified in these two subpopulations and overlapped with the TRD regions detected in the BILs (Supplementary Table S8). Among them, 9 and 11 regions were detected in SubN and SubY, respectively. Six regions (*TRD1.1*, *TRD3.1*, *TRD3.2*, *TRD6.1*, *TRD8.3*, and *TRD12.1*) were identified in common in both the subpopulations, suggesting that they were not affected by the effect of *TRD1.3*. However, there were five regions (*TRD1.2*, *TRD4.1*, *TRD5*, *TRD6.2*, and *TRD8.1*) that could only be identified in SubY, but not in SubN (Figure 4 and Supplementary Table S8), implying that these five regions may interact with *TRD1.3.* These results indicate that genetic interactions play a role in affecting TRD in the BILs.

**FIGURE 5:**
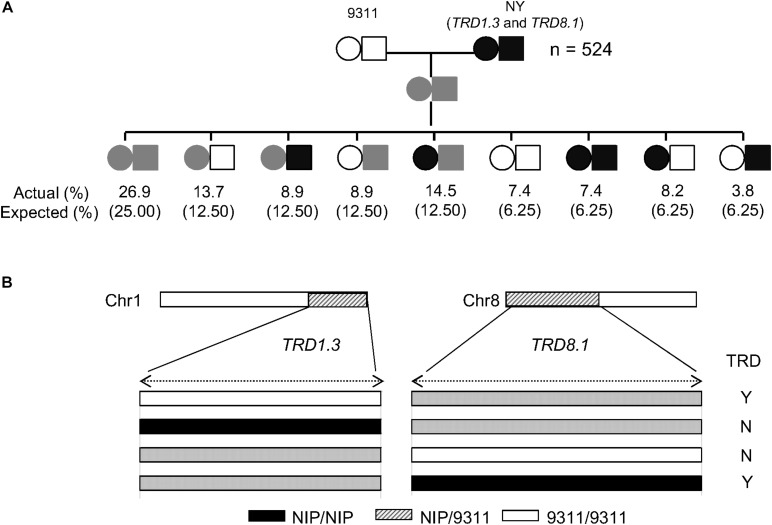
Validation of the epistatic interaction between *TRD1.3* and *TRD8.1*. **(A)** Segregation ratio of the nine genotypes at *TRD1.3* and *TRD8.1* in the F_2_ population (*n* = 524). “NY(*TRD1.3/TRD8.1*)” represent the line harboring two introduced NIP segments surrounding *TRD1.3* and *TRD8.1* within the 9311 background. The circle and rectangle indicate *TRD1.3* and *TRD8.1*, respectively. The white and black represent the homozygous 9311 and NIP genotype, respectively. The gray (streak) represents the heterozygous genotype. “Actual (%)” and “expected (%)” represent actual segregation ratio and theoretical Mendelian segregation ratio for nine genotypes. **(B)** Schematic diagram of the *TRD1.3* and *TRD8.1* interaction. “N” and “Y” indicate absence and presence of significant TRD effect in a given heterozygous region. The white, black, and gray bars represent homozygous genotypes of 9311 and NIP, and heterozygote, respectively.

As *TRD8.1* was the most significant region among the mentioned five regions that were identified only in SubY and was validated in CSSL-derived populations of both 9311 and ZS97 backgrounds (Figures 2, 3A,B), it was interesting to determine whether there was an interaction between *TRD1.3* and *TRD8.1.* One line that carries two introduced NIP segments containing both TRD regions was selected and crossed with 9311 to generate additional F_2_ population. In this F_2_ population (*n* = 524), two markers (SD1C and SD8C), tightly linked with *TRD1.3* and *TRD8.1*, respectively, were used to classify the nine genotypes. The nine genotype ratios did not fit the expected Mendel’s segregation laws in this F_2_ population (Figure 5A), suggesting that the two regions had a significant digenic interaction (χ^2^ = 24.1; *P* = 2.2 × 10^–3^). Moreover, the two alleles (NIP and 9311) of *TRD8.1* were equally transmitted to the progeny when *TRD1.3* carried the NIP allele, but *TRD8.1* had a significant effect on TRD given that *TRD1.3* had the 9311 allele (Figure 5B and Supplementary Table S9). These results confirmed that *TRD1.3* and *TRD8.1* had significant interaction effects on TRD in the segregation populations.

## Discussion

Identification and characterization of the TRD regions are essential for better understanding of the genetic basis of TRD. In the present study, we have identified 18 TRD regions in which the alleles segregated abnormally in the BIL population that was derived from the intersubspecific cross of NIP and 9311. Among them, 12 regions (75.1%) were significantly skewed toward the *indica* (9311) alleles, suggesting that the gametes carrying the *indica* alleles were transmitted at higher frequencies to the progeny than the *japonica* (NIP) alleles. These findings are consistent with previous results that the majority of *indica* alleles are transmitted as the favored alleles in intersubspecific crosses (Wang et al., 2009; Reflinur et al., 2014).

We used the CSSLs that contained a particular *japonica* (NIP) segment within two *indica* backgrounds (9311 and ZS97) to further test the TRD effects (Supplementary Tables S4, S5). Using NY-derived segregating populations, 6 out of 12 regions were validated, whereas 3 out of 9 regions were confirmed using NZ-derived segregating populations (Figure 2). There were three TRD regions (*TRD4.1*, *TRD5*, *TRD8.1*) in the 9311/NIP BILs consistently validated in both the two *indica* backgrounds, in which the different type of alleles preferentially transferred to the progeny, indicating complex mechanisms across intersubspecific hybrids (Supplementary Tables S4, S5). In addition, by comparing the regions detected in the BILs with those identified in the MH63/ZS97 F_2_ population (Li et al., 2017), we found that only *TRD4.1* was colocalized in the overlapping region of *Sd4*, suggesting that *TRD4.1* was robust in both inter- and intrasubspecific populations.

It is notable that some TRD regions detected in the BILs were not fully validated in CSSL-derived populations (Figure 2). For example, six genomic regions of *TRD1.3*, *TRD2.1*, *TRD2.2*, *TRD3.1*, *TRD3.2*, and *TRD12.2* showed significant effects on TRD in the BILs, but could not be validated in their corresponding CSSL-derived F_2_ populations. These results suggest that epistatic interaction hidden in genetic backgrounds could result in allelic frequency alteration as reported previously (Leppala et al., 2013; Li et al., 2017). We found that *TRD1.3* exhibited a significant epistatic interaction with *TRD8.1* (Figure 5). Moreover, a different allele-transmitted ability was observed in *TRD1.3* and *TRD8.1*. The NIP and 9311 alleles of *TRD8.1* were equally transmitted to the progeny if *TRD1.3* had the NIP allele, but the 9311 alleles at *TRD8.1* were preferentially transmitted to the progeny when *TRD1.3* was the 9311 allele. However, the NIP alleles at *TRD1.3* was preferentially transmitted to the progeny given that *TRD8.1* was the NIP allele, but the two alleles (NIP and 9311) of *TRD1.3* were equally transmitted to the progeny given that *TRD8.1* was the 9311 allele (Supplementary Table S9). In this case, the *japonica* alleles at *TRD8.1* could help the *japonica* alleles at *TRD1.3* have a higher chance of being transmitted to the progeny. This finding suggests that many specific TRD alleles could positively contribute to breaking intersubspecific reproductive barriers.

By comparing the TRD regions detected in this study with the ones from previous studies, we found that at least 12 regions were colocalized in the same or overlapping regions that harbored genes/loci associated with TRD or hybrid sterility (Wan et al., 1996; Li et al., 2017, 2019; Shen et al., 2017) (Figure 1 and Supplementary Table S3). Six regions (*TRD1.1*, *TRD1.3*, *TRD4.1*, *TRD6.1*, *TRD12.1*, and *TRD12.3*) were mapped in the same regions reported previously for segregation distortion (Mizuta et al., 2010; Li et al., 2017). Intriguingly, two regions (*TRD1.1* and *TRD6.1*) were located in the same regions covering the known genes *DPL1* and *DPL2* in which divergent alleles were involved in the allele TRD by pollen incompatibility in rice hybrids (Mizuta et al., 2010). *TRD3.1* in peak Bin879 (8.67–8.82 Mb) is located surrounding the *Sc* gene that has been reported to affect allele TRD (Shen et al., 2017). The other six regions (*TRD2.1*, *TRD3.1*, *TRD4.2*, *TRD8.2*, and *TRD12.1*) contain previously reported QTLs/genes for hybrid sterility that lead to reproductive barriers (Supplementary Table S3) (Wan et al., 1996; Long et al., 2008; Mizuta et al., 2010; Li et al., 2017; Shen et al., 2017). *TRD1.2* with peak Bin233 (23.38–23.47 Mb) was mapped around the two adjacent genes *SaM* and *SaF* that are involved in *indica*/*japonica* hybrid male sterility (Long et al., 2008). Therefore, the mechanisms underlying hybrid sterility also caused different probabilities of allelic transmission. In addition, we also identified six novel regions (*TRD2.2*, *TRD3.2*, *TRD5*, *TRD8.1*, *TRD8.3*, and *TRD12.2*) that have not been reported before (Supplementary Table S3). In particular, *TRD2.2* was found in peak Bin654 (approximately 200 kb) on chromosome 2. *TRD3.2* is located in peak Bin910 (12.33–12.56 Mb) with an approximately 200-kb size. *TRD5* was found in peak Bin1676 (approximately 100 kb) on chromosome 5. *TRD8.1* was located in peak Bin2207 (8.32–8.38 Mb) with an approximately 69-kb size. These novel TRD regions with a fine resolution can be further functionally exploited to improve the utilization of subspecific hybrid breeding programs.

Transmission ratio distortion can result from gametic or zygotic selections. We found that the allele frequencies at six regions (*TRD1.2*, *TRD4.1*, *TRD5*, *TRD6.2*, *TRD8.1*, and *TRD8.3*) in the relevant CSSL-derived F_2_ populations were significantly skewed toward NIP or 9311 allele, indicating that the gametic factors were involved in these regions. However, the heterozygous genotypes at the six regions in the F_2_ populations all showed the normal segregation ratio (approximately 0.5), and the observed frequency of the three genotypes exhibited no significant difference compared with the theoretical genotype frequency (Supplementary Tables S4, S5). These results indicate that the gametic factor is involved in the TRD regions. Meanwhile, other six regions (*TRD1.3*, *TRD2.1*, *TRD2.2*, *TRD3.1*, *TRD3.2*, and *TRD12.2*) in corresponding F_2_ populations showed normal allele segregation ratios (Supplementary Tables S5, S6), which did not provide enough information to distinguish gametic or zygotic selections.

## Conclusion

Eighteen TRD regions were identified in the BIL population that was derived from the intersubspecific cross of 9311 and NIP with a high-density SNP map. Among them, three TRD regions were validated in CSSL-derived secondary populations within both the two *indica* genetic backgrounds. Furthermore, digenic interaction between *TRD1.3* and *TRD8.1* was found affecting TRD. The identification of the TRD regions can be utilized in further map-based cloning of the causal genes for TRD, which will facilitate understanding of the genetic basis of reproductive barriers. In addition, our data reveal that TRD, in some cases, can positively contribute to breaking the intersubspecific reproductive barrier, therefore providing an attractive strategy of introgression breeding in intersubspecific hybrids in rice.

## Data Availability Statement

The datasets generated for this study can be found in DRYAD 10.5061/dryad.sf7m0cg44.

## Author Contributions

SY designed and conceived the research. CZ, DW, LT, QS, ZY, XT, and JW developed the population and NILs. CZ and DW conducted the experiments. CZ and JW analyzed the data. CZ, HH, and SY wrote the manuscript. All the authors read and approved the final manuscript.

## Conflict of Interest

The authors declare that the research was conducted in the absence of any commercial or financial relationships that could be construed as a potential conflict of interest.

## Footnotes

1 http://rice.plantbiology.msu.edu/index.shtm

2 http://www.r-project.org/

## Supplementary Material

Supplementary Figure S1 Allele frequencies at the bins in backcross inbred lines (BILs). The cyan and orange colors indicate 9311 and Nipponbare alleles, respectively. The *x*-axis represents the physical location of the bins along each numbered chromosome. The *y*-axis represents the allele frequencies. Horizontal dashed lines indicate the theoretical allele segregation ratio (3:1) in the BILs.

Supplementary Table S1 Bin genotypes of the backcross inbred line population derived from the cross of 9311 and Nipponbare.

Supplementary Table S2 The number of bins with allele transmission ratio distortion in the backcross inbred line population derived from the cross of 9311 and Nipponbare.

Supplementary Table S3 Genomic regions detected for allele transmission ratio distortion in the backcross inbred lines.

Supplementary Table S4 Twelve transmission ratio distortion regions tested in NY-derived populations in the 9311 background.

Supplementary Table S5 Validation of nine transmission ratio distortion regions in NZ-derived populations in the ZS97 background.

Supplementary Table S6 Validation of five transmission ratio distortion regions in MZ-derived populations in the ZS97 background.

Supplementary Table S7 Primers used for genotyping F_2_ populations.

Supplementary Table S8 The TRD regions detected for allele transmission ratio distortion in the two subpopulations.

Supplementary Table S9 Epistatic interaction of *TRD1.3* and *TRD8.1* regions in the F_2_ population derived from NY(*TRD1.3*/*TRD8.1*) and 9311.
