# Supplementary figures and images for: Genetic Dissection and Validation of Chromosomal Regions for Transmission Ratio Distortion in Intersubspecific Crosses of Rice
Source: Front Plant Sci. 2020 Oct 27;11:563548. doi: 10.3389/fpls.2020.563548 (PMC7655136; doi:10.3389/fpls.2020.563548)

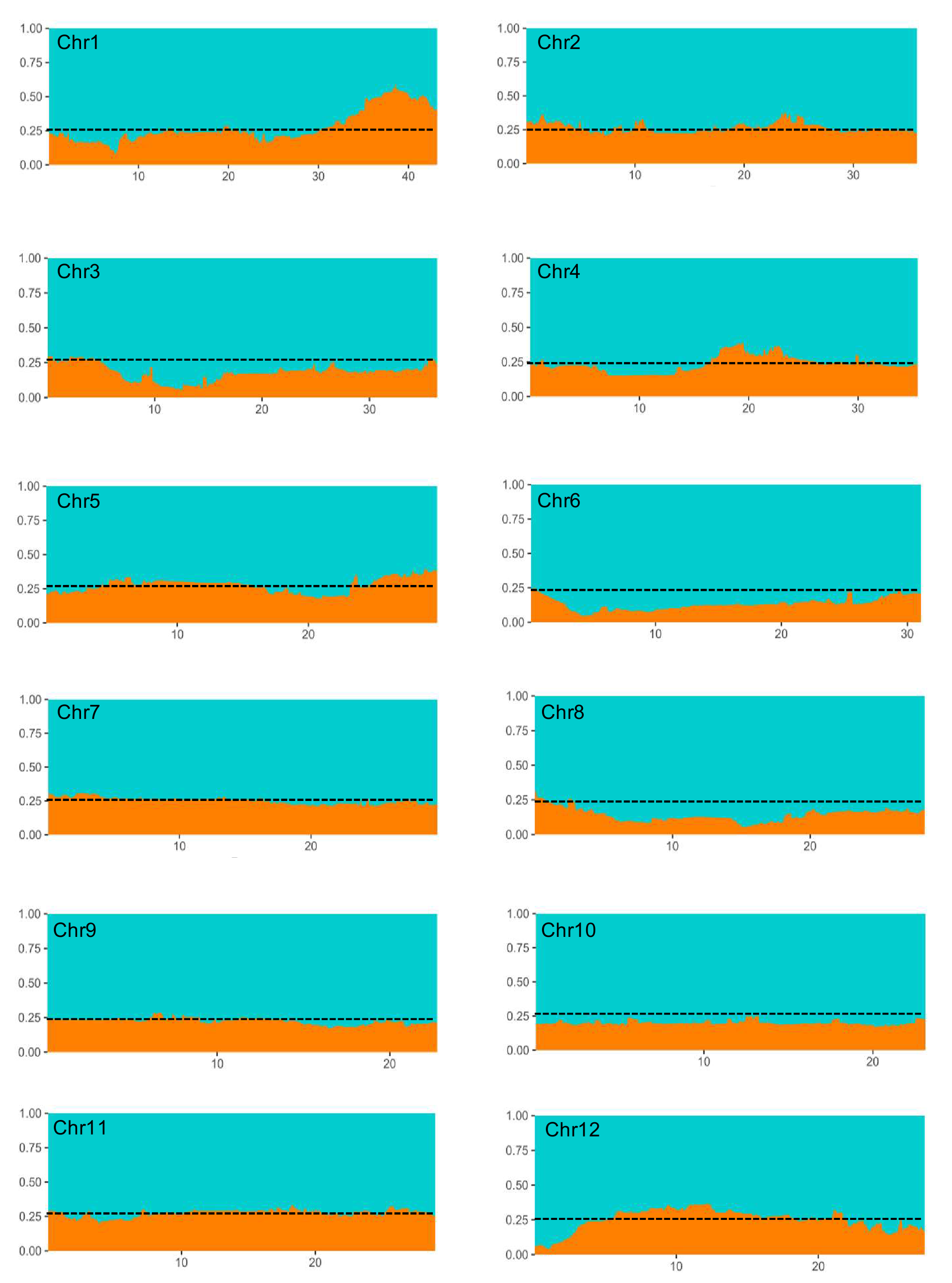

Supplement: Supplementary Figure S1 — Allele frequencies at the bins in backcross inbred lines (BILs). The cyan and orange colors indicate 9311 and Nipponbare alleles, respectively. The x-axis represents the physical location of the bins along each numbered chromosome. The y-axis represents the allele frequencies. Horizontal dashed lines indicate the theoretical allele segregation ratio (3:1) in the BILs. [file Image_1.TIF]
